# Non-insulin diabetes medicines: a narrative review for anaesthetists and intensivists

**DOI:** 10.1016/j.bja.2025.05.050

**Published:** 2025-07-11

**Authors:** Sarah Tinsley, Claire Frank, Daniel Stubbs, Ketan Dhatariya, Roger D. Knaggs, Nicholas A. Levy

**Affiliations:** 1 Pharmacy Department, University Hospitals of North Midlands NHS Trust, Stoke-on-Trent, UK; 2 Pharmacy Department, Wrexham Maelor Hospital, Betsi Cadwaladr University Health Board, Wrexham, UK; 3 Centre for Perioperative Care Fellow; 4 Perioperative, Acute, Critical, and Emergency Care Section, University of Cambridge, Cambridge, UK; 5 Department of Anaesthesia, Cambridge University Hospitals NHS Foundation Trust, Cambridge, UK; 6 Elsie Bertram Diabetes Centre, Norfolk and Norwich University Hospitals NHS Foundation Trust, Norwich, UK; 7 University of East Anglia Medical School, Norwich, UK; 8 School of Pharmacy, Pain Centre Versus Arthritis, University of Nottingham, Nottingham, UK; 9 Department of Anaesthesia and Perioperative Medicine, West Suffolk NHS Foundation Trust, Suffolk, UK

**Keywords:** biguanide, diabetes mellitus, incretins, insulin secretagogue, perioperative, sodium glucose co-transporter 2 inhibitor, sulfonylureas, thiazolidinedione

## Abstract

Diabetes mellitus is characterised by an increased blood glucose concentration. Over the past two decades, multiple new agents have emerged to help treat the condition, of which several classes of agent have been shown to reduce the risk of cardiovascular morbidity and mortality. In addition, there have been several developments in the pharmacology of non-insulin diabetes medicines, working on different aspects of gut and renal glucose absorption, hepatic metabolism, insulin sensitivity, and incretin hormone physiology. This narrative review focuses on these non-insulin diabetes medicines. We discuss the different classes, including insulin secretagogues, biguanides, alpha-glucosidase inhibitors, thiazolidinediones, sodium glucose co-transporter 2 (SGLT2) inhibitors, and the newer incretin-based therapies. Each section focuses on the mode of action but also considerations relevant to anaesthetists, surgeons, and other members of the perioperative team caring for people with diabetes mellitus receiving these agents to ensure safe care and avoidance of complications.

**Editor’s key points**

• Appropriate perioperative manipulation of non-insulin, glucose-lowering medications allows for day-of-surgery admission, reduced dysglycaemia, and shortened stay. Individualised management of these medicines remains a challenge.
• This review summarises the pharmacology of various non-insulin, glucose-lowering agents and details how perioperative dose adjustments can help to minimise dysglycaemia.
• Nonspecialists need to familiarise themselves with the perioperative manipulation of non-insulin, glucose-lowering medicines, particularly with the increased use of incretin-based drugs and SGLT2 inhibitors.

Type 2 diabetes mellitus (T2DM) is one of the most common metabolic diseases worldwide. Although potentially preventable, the prevalence of T2DM is rising worldwide owing to a strong association with obesity and a sedentary lifestyle and thus represents a significant public health burden.^1^ A recent estimate suggested that around one in nine adults around the world (828 million) has diabetes.^2^ Further, more people with diabetes are referred for, or have, an operation compared with those without diabetes.^3^^,^^4^

T2DM is often managed with lifestyle and dietary changes, and then non-insulin pharmacological interventions are instituted with insulin reserved for resistant T2DM.^5^ Non-insulin medicines licensed for treating T2DM have various modes of action, including stimulating insulin secretion, altering gastrointestinal absorption, altering renal reabsorption of glucose, and reducing insulin resistance.^6^

This narrative review discusses the non-insulin medicines used for the treatment of T2DM, their pharmacology, and their recommended management both in the perioperative period and within critical care. There is a parallel publication on the perioperative use of insulin.^7^ In addition, interested readers are directed to further publications on the perioperative use of a variable rate i.v. insulin infusion (VRIII).^8^^,^^9^

## Non-insulin regimens for people with type 2 diabetes

Non-insulin diabetes medicines are used as first-line options in the treatment of T2DM, but almost none are licensed for use in people with type 1 diabetes mellitus (T1DM). However, in the USA, an amylin analogue, pramlintide, and in Japan, sodium glucose co-transporter-2 inhibitors (SGLT2is) are available as adjunctive treatments alongside insulin for T1DM.

The medications used to treat T2DM used to be called ‘oral hypoglycaemic medicines’; however, most of the incretin agonists require subcutaneous injection, and only sulfonylureas and meglitinides cause hypoglycaemia. Therefore, use of the term ‘oral hypoglycaemic medicines’ is no longer recommended.

Pharmacological treatment should be combined with lifestyle changes such as weight management and increased physical activity, both of which can improve insulin resistance.^5^ Non-insulin medicines are preferable to insulin in individuals who may be more susceptible to hypoglycaemia, or who may be reliant on others to administer the subcutaneous injection or may have reduced adherence with injectable formulations.

Available treatments include sulfonylureas, alpha-glucosidase inhibitors, biguanides (i.e. metformin), SGLT2is, glucagon-like peptide-1 receptor agonists (GLP-1RAs), and dipeptidyl peptidase-4 (DPP-4) inhibitors, many of which are available as combination products. The recommended first-line treatment is usually metformin, with addition of SGLT-2i for patients with proven/high risk of developing cardiovascular disease. Other medicines are considered when metformin is contraindicated or not tolerated, or as sequential or alternative treatments in people who are not achieving optimal glycaemic control.^5^ ^10^ Progressive deterioration in insulin secretion can result in treatment failure requiring the introduction of insulin when non-insulin options have been exhausted.^11^

## Development of non-insulin diabetes medicines

Initial non-insulin pharmacological options included sulfonylureas and biguanides. Sulfonylureas developed from the observation that hypoglycaemia occurred in patients receiving sulphur-containing antibiotics.^12^ Whereas early sulfonylureas were long-acting and have now been discontinued because of the risk of hypoglycaemia, second-generation sulfonylureas have a shorter duration of action and are still in routine use.^13^ The debate on whether they should still be used in routine clinical practise given their lack of cardiovascular benefit and potential to do harm is outside the scope of the current manuscript.

Guanidine, extracted from *Galega officinalis*, a medieval herbal remedy for diabetes, was a precursor of biguanides.^14^ Both guanidine and the early biguanides phenformin and buformin were withdrawn because of hepatotoxicity and increased lactic acidosis risk, but metformin is still widely used as the first-line treatment for type 2 diabetes.^5^

**Fig 1:**
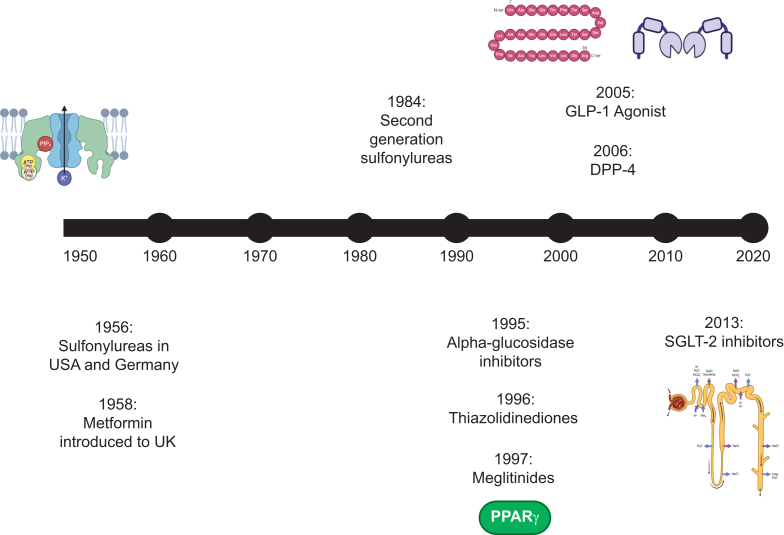
Timeline for development of non-insulin diabetes medicines. Created in BioRender. DPP-4, dipeptidyl peptidase-4; GLP-1, glucagon-like peptide-1; PPARγ, *peroxisome proliferator-activated receptor-*γ*;* SGLT-2, sodium glucose co-transporter 2.

In the 1990s, alpha-glucosidase inhibitors, thiazolidinediones, and meglitinides were marketed, although their use has largely been superseded in recent years by the availability in treatment options which, in large randomised clinical trials, have been shown to have cardiovascular and renal benefits (Fig. 1). SGLT-2i first came onto the market in 2013, when canagliflozin was approved for use. Since then, four other SGLT-2i medicines have been introduced.^15^^,^^16^ These drugs are also now licensed for people without diabetes because they have been shown to be beneficial in reducing adverse cardiovascular outcomes in people with heart failure and helping to prevent the progression of chronic renal disease.

The importance of GLP-1 and its potential importance in T2DM were first described in the early 1990s. Endogenous GLP-1 has a very short half-life (1–2 min), because it is broken down by the enzyme DPP-4. Because of the short half-life of GLP-1, agents that were resistant to the action of DPP-4, or DPP-4 inhibitors, were then developed. In 2005, the first of these GLP-1RAs, exenatide, was marketed. There are currently five GLP-1RA medicines available.^15^^,^^16^ The newer agent tirzepatide is a dual GLP-1RA and glucose-dependent insulinotropic polypeptide (GIP) receptor agonist and became available for the treatment of T2DM in 2022 in the USA and 2024 in the UK.

The timeline for the development of non-insulin diabetes medicines is summarised in Figure 1.

## Perioperative modification of non-insulin treatment for type 2 diabetes

During surgery, it is essential to prevent dysglycaemia (both hypo- and hyperglycaemia). Previously, most people requiring surgery were managed with i.v. insulin with cessation of their normal diabetes medicines. This exposed them to the risks of i.v. insulin and denying day surgery.^17^ Current practise now supports dose modification of normal medicines when safe; however, there are well-known risks associated with non-insulin diabetes medicines in people undergoing surgery, but these differ depending on the specific mechanism of action of each agent. These risks include (but are not limited to) hypoglycaemia (sulfonylureas and meglitinides)^13^; euglycaemic diabetic ketoacidosis (DKA) (SGLT2i)^18^; possible delayed gastric emptying (GLP-1RA)^19^; and lactic acidosis (metformin in the presence of renal failure).^20^ The risks and benefits of continuation/cessation of each class of medicine are summarised in Table 1.

**Table 1:** Summary of the controversies surrounding perioperative continuation of non-insulin diabetes medicines. The GLP-1/GIP RA agonists became available in the early 2020s. GLP-1RA, glucagon-like peptide-1 receptor agonists; GLP-1/GIP RA, glucagon-like peptide-1/glucose-dependent insulinotropic polypeptide receptor agonist; SGLT2, sodium glucose-like transporter-2 inhibitors; DPP-4, dipeptidyl peptidase 4 inhibitors; PONV, postoperative nausea and vomiting; ADA, American Diabetes Association; FDA, Food and Drug Administration; CPOC, Centre for Perioperative Care; SAMBA, Society for Ambulatory Anesthesia; JBDS, Joint British Diabetes Societies for Inpatient Care. ∗Omitting more than two doses of GLP-1RAs or GLP-1/GIP RAs means that reintroduction must start from the lowest doses again, which will prolong glycaemic variability/length of bridging therapy required.

Class	Benefits of continuing	Risks of continuing	Risks of stopping	Alternatives to continuing	UK product license^21^	US product license/recommendations^21^,^22^	UK Guidance (CPOC and JBDS) ^23^,^24^
Sulfonylureas e.g. gliclazide, glimepiride, glipizide	Not safe to continue	Hypoglycaemia	Risks of perioperative hyperglycaemia and the ensuing complications, including infection, acute coronary syndromes, acute kidney injury, cerebrovascular accidents, and death Requirement for variable rate i.v. insulin infusion/sliding scale insulin and consequent risk of their use Risk of longer stay in hospital for glycaemic control/treatment of perioperative complication Risk of cancellation on day of surgery because of suboptimal glycaemic control	Bridging with insulin therapy with the inherent risks of hypoglycaemia and suboptimal glycaemic control	No recommendation made	Omit on the morning of surgery (ADA)	If morning surgery, omit morning dose If afternoon surgery, omit all doses due
Meglitinides e.g. repaglinide	Not safe to continue	Hypoglycaemia			No recommendation made	Omit on morning of surgery (ADA)	If morning surgery, omit morning dose if not eating If afternoon surgery, take morning dose as normal if eating
Alpha-glucosidase inhibitors e.g. acarbose	Facilitates perioperative control of diabetes. Prevents routine requirement for variable rate i.v. insulin infusions/sliding scale	Low risk of hypoglycaemia, particularly when used in combination with other agents			No recommendation made	No recommendation made	Omit on the day of surgery if not eating
Thiazolidinediones e.g. pioglitazone	Facilitates perioperative control of diabetes. Prevents routine requirement for variable rate i.v. insulin infusions/sliding scale	None			No recommendation made	No recommendation made	Take as normal
Biguanides i.e. metformin	Facilitates perioperative control of diabetes. Prevents routine requirement for variable rate i.v. insulin infusions/sliding scale	Concern over the risk of lactic acidosis (although largely unsubstantiated)			Omit at the time of surgery and restart 48 h after operation	Hold on the day of surgery (ADA) Continue unless renal impairment (SAMBA)	If taken once or twice a day—take as normal If taken three times per day, omit the lunchtime dose
GLP-1RA∗ e.g. exenatide, dulaglutide, liraglutide, semaglutide GLP-1/GIP RA∗ tirzepatide	Facilitates perioperative control of diabetes. Prevents routine requirement for variable rate i.v. insulin infusions/sliding scale	Concern over delayed gastric emptying and complications of risk of aspiration and PONV			No specific recommendation made but consider increased risk of residual gastric content before performing procedures with general anaesthesia or deep sedation	No data to be able to make a recommendation (ADA)	Take as normal
DPP-4 inhibitors e.g. alogliptin, linagliptin, saxagliptin, sitagliptin, vildagliptin	Facilitates perioperative control of diabetes. Prevents routine requirement for variable rate i.v. insulin infusions/sliding scale	None			No recommendation made	Withhold on the morning of surgery	Take as normal
SGLT2 inhibitors e.g. empagliflozin, dapagliflozin, canagliflozin, ertugliflozin	Not safe to continue. These agents must not be continued or initiated in unwell inpatients	Euglycaemic DKA, with inpatient safety not being established^12^			No specific recommendation made but use with caution in conditions that lead to restricted food intake or severe dehydration and those with increased insulin requirements due to surgery	Consider stopping 3 days (ertugliflozin 4 days) before scheduled surgery (FDA and ADA)	Omit on day before surgery and on day of surgery

This narrative review discusses the pharmacology of non-insulin diabetes medicines, so that optimal decisions on perioperative cessation/and dose modification can be made.

In all instances, consideration should be given to prescribing the components of combination products (e.g. Xigduo®, AstraZeneca (London) containing dapagliflozin and metformin) as separate medicines perioperatively to allow for individualised management of each component.

## Sulfonylureas

Sulfonylureas are insulin secretagogues and are the oldest class of oral non-insulin medicines being first used in the 1950s.^25^ They bind to the sulfonylurea receptor on the cell membrane of pancreatic beta-cells, leading to stimulation of insulin secretion by inhibition of the potassium-adenosine triphosphate (K-_ATP_) channel. They require functioning beta-cells to increase insulin production.^25^ ^26^ As a result of exerting their effects independently of blood glucose levels, the risk of hypoglycaemia is high.^25^ ^26^ This can be a clinical issue in patients who are fasting in preparation for surgery.^18^ First-generation sulfonylureas (e.g. chlorpropamide and glibenclamide) were very long lasting and were associated with the greatest risk of hypoglycaemia. Second-generation agents (e.g. gliclazide, glipizide, and glimepiride) are shorter acting (although some are available as extended-release formulations) but have a lower risk of hypoglycaemia.

### Perioperative management of sulfonylureas

Overall, there is limited evidence available on the perioperative management of sulfonylureas.^18^ However, because of the risk of hypoglycaemia, sulfonylureas are generally recommended to be withheld on the morning of surgery.^18^ ^23^^,^^27^, ^28^, ^29^, ^30^ It has been proposed that they should be continued when the incidence of hypoglycaemia is low, for example, when insulin or other glucose-lowering agents are not used, or when sulfonylureas have been used for longer than 3 months. However, because of the small sample size, the studies supporting this have not had the power to fully address the clinical significance.^18^ ^31^

Individuals undergoing surgery that requires a liver reduction diet (LRD) or those that require bowel preparation may need a longer cessation period that would coincide with reduced food intake. They should be restarted once the person is eating and drinking normally and once the VRIII, if used, has stopped.^23^ ^28^ Canadian guidelines recommend starting at 50% of the person’s usual dose if they are eating at 80% of normal, and only restarting if their renal function is at baseline.^29^

After bariatric surgery, people with T2DM may have a rapid decrease in their blood glucose levels, meaning sulfonylurea use may need to be reviewed or even stopped, especially because of the potential risk of hypoglycaemia.^32^

## Biguanides

The biguanides have been in clinical use for more than 60 yr.^26^ They reduce hepatic glucose production, increase insulin sensitivity, and delay intestinal glucose absorption but do not stimulate insulin secretion or contribute to hypoglycaemia.^25^ Most early biguanides were withdrawn because of their toxic side-effects, including lactic acidosis. However, metformin remains the first-line treatment for most adults with T2DM either as monotherapy or in combination with other agents.^10^^,^^26^

### Perioperative management of biguanides

Historically, metformin has been withheld before operation, because of concerns about metformin-associated lactic acidosis (MALA). These concerns are a legacy of the lactic acidosis caused by earlier biguanides. Metformin differs from these earlier biguanides in both molecular structure and pharmacokinetics.^33^ Furthermore, a Cochrane review concluded there was no evidence metformin is associated with increased lactate levels or risk of lactic acidosis, compared with other treatments.^33^

However, the manufacturers still advise that metformin should be discontinued for all patients at the time of surgery under general, spinal, or epidural anaesthesia, or when receiving iodinated contrast agents and gadolinium contrast administration for MRI, and restarted no earlier than 48 h after, on resumption of oral nutrition and where renal function has been checked and is stable—that is, a creatinine that is <135 μmol L^−1^ or an estimated glomerular filtration rate (eGFR) >30 ml/min/1.73 m^2^. Similarly, the Australian and Canadian guidelines recommend that metformin (and all non-insulin medicines) should be held on the day of surgery, even though they recognise that some of these medicines may be safe to continue.^28^^,^^29^

In a multicentre study, continuation of metformin before elective noncardiac surgery did not improve glucose control, when compared with patients who stopped it 24 h before surgery.^34^ Of note, participants in the randomised controlled trial had excellent preoperative glycaemic control, and nearly half underwent gastric bypass surgery, so may not be representative of the wider population. However, importantly, any increase in lactate levels was not clinically relevant.^34^ Likewise, metformin continuation was not associated with an increased risk of adverse outcomes in patients undergoing cardiac surgery.^20^^,^^35^ Indeed, studies have demonstrated potential benefits from perioperative metformin use, including a lower incidence of postoperative acute kidney injury after cardiac and noncardiac surgery,^36^^,^^37^ complications after arthroplasty,^38^ and complications and 30-day mortality after major surgery,^39^ but no statistically significant decrease in incidence of postoperative delirium.^37^

Pragmatically, given the low incidence of MALA, some organisations, including the Centre for Perioperative Care (CPOC),^23^ the Joint British Diabetes Societies,^24^ and the Society for Ambulatory Anesthesia (SAMBA),^30^ advocate for continuation of metformin (with the exception of any lunchtime doses) unless those with chronic kidney disease require contrast media.^23^^,^^30^ Metformin can be continued before bariatric surgery; even in patients requiring LRD, providing capillary blood-plasma glucose (CBG) is closely monitored. After bariatric surgery, there may also be benefits of continuing metformin, potentially at a reduced dose, although modified-release preparations will require changing to immediate-release preparations to facilitate absorption.^40^

## Alpha-glucosidase inhibitors

Acarbose and voglibose inhibit alpha glucosidase, which is involved in intestinal glucose absorption. Because they stop glucose increase by reducing post-prandial hyperglycaemia, the risk of hypoglycaemia is very low because their glucose-lowering efficacy is dependent on carbohydrate intake.^26^ However, in the UK, acarbose is no longer recommended as a treatment option in clinical guidelines so is not widely used.^10^ Voglibose is available in other parts of the world and continues to be used.

### Perioperative management of alpha-glucosidase inhibitors

These drugs should be withheld before morning surgery, but the morning dose can be continued if eating breakfast before afternoon surgery. Once normal enteral intake resumes, acarbose can be restarted.^26^^,^^23^^,^^30^ However, prescribers should be cognizant that the side-effects include flatulence and diarrhoea, which may be relevant after operation.^25^^,^^30^ For patients undergoing bariatric surgery, these agents should be discontinued when LRD commences, CBG monitored, and the ongoing need for treatment reviewed after operation.^40^

## Thiazolidinediones

Thiazolidinediones increase insulin sensitivity in liver, adipose tissue, and skeletal muscle by activating peroxisome proliferator-activated receptor-γ, a nuclear transcription factor.^25^^,^^28^ Pioglitazone, the only thiazolidinedione available in the UK, is indicated as one of several options if metformin use is contraindicated.^10^ However, use has reduced in recent years after concerns about exacerbation of heart failure and the unsubstantiated risk of bladder cancer.^25^^,^^41^

### Perioperative management of thiazolidinediones

Thiazolidinediones do not cause hypoglycaemia, but some international guidelines recommend all non-insulin medicines are withheld on the day of surgery, even if only as a precaution.^28^^,^^29^ However, pragmatically, because of the length of time it takes to work, many guidelines recommend that pioglitazone can be continued before operation.^26^^,^^23^^,^^30^ Pioglitazone can also be continued before bariatric surgery, even in patients requiring LRD, but the ongoing need for treatment should be reviewed after operation.^40^

## Meglitinides

Meglitinides have the same mechanism of action as sulfonylureas, in that they stimulate insulin release by inhibiting the K-_ATP_ channel. They are more rapid and short-acting than sulfonylureas.^25^^,^^26^ Repaglinide is the only currently available meglitinide in the UK and is indicated either alone or in combination with metformin for the treatment of T2DM.^42^ However, it is no longer recommended as a treatment option in clinical guidelines and is not widely used.^10^ Hypoglycaemia can occur with meglitinides, although the risk appears to be less than sulfonylureas.^25^ This needs to be considered for patients who are fasting in preparation for surgery.

### Perioperative management of meglitinides

Because of its short duration of action, management of repaglinide in the UK is dependent on the time of surgery and the period of fasting. For procedures that occur in the morning where patients will be fasting from the night before, morning doses of meglitinides should be omitted. For procedures in the afternoon, morning doses of meglitinides should be taken if the patient is eating.^23^^,^^40^ Individuals undergoing surgery that requires an LRD or those that require bowel preparation may need a longer cessation period that would coincide with reduced food intake.^23^^,^^40^

American, Australian, and Canadian guidelines make no differentiation on management for the timing of surgery or fasting period and recommend that meglitinides should be held on the day of surgery.^26^^,^^27^, ^28^, ^29^, ^30^ In the ambulatory setting, it may be possible to continue meglitinides,^30^^,^^31^ although there is limited evidence base to inform this practise currently and guidelines for this surgical population recommend that they should be omitted.^26^

Postoperative resumption of meglitinides is recommended once the patient is eating and drinking normally and, if applicable, once the VRIII has stopped.^28^^,^^30^^,^^40^ Canadian guidelines recommend starting at 50% of the patient’s usual dose if they are eating at 80% of normal, and only if the patient's renal function is at baseline.^29^

After bariatric surgery, people with T2DM may have a rapid decrease in their blood glucose levels, meaning these agents may need to be reviewed or even stopped.^32^

## Incretin agonists (glucagon-like-peptide-1 receptor agonists and glucose-dependent insulinotropic polypeptide agonists)

The incretin hormones, GLP-1 and GIP, are secreted from the gut wall in response to dietary carbohydrate intake. They increase insulin secretion through pancreatic beta cell stimulation, reduce glucagon secretion through alpha cell inhibition, increase satiety, and delay gastric emptying.^43^ The glucose-dependent effect of incretin hormones minimises the risk of hypoglycaemia.^44^ Endogenous GLP-1 is rapidly hydrolysed and degraded by DPP-4 and so is unsuitable as a pharmacological agent. These hormones have been modified to allow them to have long half-lives, resistant to DPP-4 degradation.

**Fig 2:**
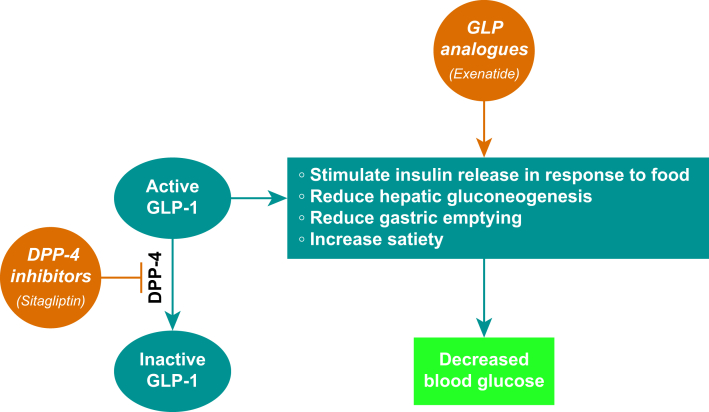
Mechanism of action of incretin agonists and DPP-4 inhibitors. DPP-4, dipeptidyl peptidase-4; GLP-1, glucagon-like peptide-1.

GLP-1 receptor agonists (dulaglutide, liraglutide, and semaglutide) and dual GLP-1/GIP receptor agonists (tirzepatide) exploit these actions for therapeutic effect (Fig. 2). All are licensed for the treatment of T2DM, but more recently, liraglutide, semaglutide, and tirzepatide have also been licensed for weight management in people without diabetes.^42^ Data suggest that 50% of all adults would be eligible for these drugs just using clinical trial inclusion criteria^45^; hence, the number of people on these agents is likely to increase significantly in the coming years. Given the multitude of other conditions these drugs are under investigation for, people without diabetes or obesity may be receiving them in the near future.^46^

### Risk of aspiration

Reduced peristalsis and delay in gastric emptying are part of the mode of action of GLP-1 and GIP agonists.^44^ Unsurprisingly there have been case reports of residual gastric content and aspiration after standard perioperative fasting periods,^47^ leading the UK Medicines and Healthcare Products Regulatory Agency to warn of potential risk of pulmonary aspiration under general anaesthesia or deep sedation.^48^ Meta-analysis of patients undergoing upper endoscopy found a statistically significant increased risk of residual gastric contents, despite standard fasting periods, in patients taking GLP-receptor agonists.^49^ Seven out of eight prospective and retrospective studies in a scoping review also reported a significant increase in residual gastric contents. Notably, confounding factors for increased residual gastric contents (e.g. diabetes, reflux, other medicines) were present in all but one case report.^50^

There is considerable heterogenicity in published studies in relation to the gastric emptying methodology, formulations, and time since last dose.^44^^,^^47^ It has been suggested that short-acting GLP-1RAs have a more pronounced effect on gastric emptying, but these are now very rarely used clinically given their lack of cardiovascular benefit compared with the newer, weekly agents.^47^ It is also thought that prolonged duration of use attenuates the effect on gastric emptying, possibly because of tachyphylaxis.^47^^,^^51^

Conversely, a meta-analysis of five studies using scintigraphy, the gold standard measure of gastric emptying, reported a 36-min delay in gastric emptying compared with placebo. Ten studies using the paracetamol absorption test, an indirect method, reported no significant delay in gastric emptying with GLP-1RAs. Subgroup analysis showed no significant difference in solid-phase and liquid-phase gastric emptying between long-acting and short-acting agents. Meta-regression found treatment duration did not have a significant impact on gastric emptying suggesting no significant tachyphylaxis.^52^ To reduce the potential risk of pulmonary aspiration, the use of point-of-care gastric ultrasound has been advocated, but it is acknowledged that this is not available in many centres.^19^^,^^53^^,^^54^

### Perioperative management of incretin agonists

On the basis of case reports or small case series of aspiration, the ASA recommended in June 2023 that daily GLP-1RAs should be withheld on the day of surgery, and weekly preparations should be withheld the week before surgery.^55^ The value of this recommendation was questioned by the UK’s CPOC and the American Gastroenterological Association.^56^^,^^57^ The CPOC suggested continuation and risk assessment, with the risk assessment dictating the choice of airway technique.

In October 2024, the ASA released new guidance reversing their original position.^58^ In agreement with other new guidance, this recommends the need for individual risk assessment, shared decision making, and generally continuation of the medicines albeit with alteration to the anaesthetic technique or the starvation period.^19^ More recently, a retrospective study has been published demonstrating that continuation of GLP-1RA prescription in patients with diabetes was associated with significant reductions in risk-adjusted readmission, wound dehiscence, and haematoma, and no difference in infection and bleeding rates.^59^ The validity of these results will need to be further assessed.

### Perioperative management of type 2 diabetes with incretin agonist regimens

It has been proposed that there may be a place for GLP-1RA in the perioperative management of people with T2DM, rather than using insulin. Liraglutide has been studied in a number of small randomised controlled trials in comparison with insulin regimens, where it has been found that patients require less insulin (i.e. less additional doses, lower doses, and reduced insulin volumes) and have more stable glycaemic control.^18^ At present, the evidence base remains limited to make any recommendations.

## Dipeptidyl peptidase-4 inhibitors

DPP-4 inhibitors are a class of medicines that also target the incretin system. DPP-4 is an enzyme, that inactivates incretin hormones; hence, DPP-4 inhibitors prolong the hypoglycaemic effects of endogenous incretin hormones by preventing their breakdown.^25^ This means that, unlike the pharmacological doses of GLP-1RA, endogenous GLP-1 concentrations are maintained at physiological concentrations (see Fig. 2).

DPP-4 inhibitors are licensed as either monotherapy (except sitagliptin) or in combination with other antidiabetic medicines (including insulin).^42^ Five DPP-4 inhibitors (alogliptin, linagliptin, saxagliptin, sitagliptin, and vildagliptin) are currently available in the UK, all entering the market after regulatory authority approval between 2006 and 2013.^60^ Fixed-dose combination preparations containing a DPP-4 inhibitor with metformin or an SGLT-2i are also available.^42^

DPP-4 inhibitors exert their effects in a glucose-dependent manner; hence, there is minimal risk of them causing hypoglycaemia. It has been shown that fewer hypoglycaemic events occurred despite blood glucose being lowered to the same concentrations as when using insulin therapy in individuals undergoing noncardiac surgery.^18^ In general, DPP-4 inhibitors have a low adverse reaction risk, although they have been associated with the (uncommon) increased risk of acute pancreatitis, which was highlighted by the UK Medicines and Healthcare Products Regulatory Agency after post-marketing reports.^61^

### Perioperative management of dipeptidyl peptidase-4 inhibitors

Irrespective of fasting duration or time of the planned procedure, UK guidance advises that DPP-4 inhibitors can be continued before operation.^26^^,^^23^^,^^30^

The exception to this is for combination products containing metformin, which may need to be reviewed in patients with an eGFR less than 60 ml/min/1.73 m^2^ if having contrast media; combination products containing SGLT-2i; and those who may be undergoing surgery that requires undertaking a LRD before operation.^40^

Conversely, American, Australian, and Canadian guidelines recommend all DPP-4 inhibitors should be withheld on the day of surgery, although there is recognition that continuation may be safe.^27^, ^28^, ^29^ A small randomised controlled trial conducted by Gasanova and colleagues,^31^ although not specifically looking at DPP-4 inhibitor continuation, found that blood glucose was lower without associated hypoglycaemia in all patients who continued their oral anti-diabetes medicines and potentially supports the UK stance for the perioperative continuation of these agents owing to their low hypoglycaemia risk. Another trial in medical and surgical inpatients randomised to sitagliptin with basal insulin compared with a basal-bolus regimen showed no differences in glucose concentrations or other outcomes, but the use of sitagliptin was shown to be less labour intensive.^62^

In the postoperative period, UK guidance recommends that DPP-4 inhibitors can be restarted once the patient has started eating and drinking normally and, if commenced perioperatively, the VRIII has been stopped.^40^ Australian guidelines agree with this approach.^28^ Conversely, Canadian guidance recommends DPP-4 inhibitors can be restarted immediately after operation, even if the patient is nil by mouth with no enteral intake.^29^

Care needs to be taken for patients taking combination products containing a DPP-4 inhibitor with metformin or an SGLT-2i, as different postoperative advice may need to be followed in respect to the component parts.^40^

After bariatric surgery, people with T2DM may have a rapid decrease in their blood glucose concentrations, meaning that even though they do not lower glucose concentrations, DPP-4 inhibitors may need to be reviewed or even stopped.^32^

The differences between these guidelines reflect that, even though these agents have been available for almost 20 yr, there remains limited good-quality evidence available to guide the most appropriate management of DPP-4 inhibitors in the perioperative period.^18^

### Perioperative management of type 2 diabetes with DPP-4 inhibitor regimens

As for GLP-1RA, there have been a couple of small-scale studies looking at the use of DPP-4 inhibitors compared with usual insulin regimens for the perioperative management of people with T2DM. One study reviewed linagliptin combined with fast-acting insulin *vs* a basal-bolus regimen; mean daily blood glucose levels were higher in the linagliptin group but they had fewer hypoglycaemic events. A further study of sitagliptin *vs* placebo use in people undergoing coronary artery bypass grafting found no statistical difference in postoperative glucose concentrations between the groups.^18^ In cardiac surgical patients, there are benefits for both long-term cardiac and cerebrovascular complications in those taking DPP-4 inhibitors compared with other anti-diabetes agents.^26^

## Sodium glucose co-transporter-2 inhibitors

### Sodium glucose co-transporter-2 inhibitor use in diabetes

The sodium glucose co-transporter-2 (SGLT-2) proteins are found in the kidneys, mainly in the proximal tubules, and play a role in renal glucose reabsorption. SGLT-2 is responsible for 90% of total glucose reabsorption. SGLT-2i therefore prevents the reabsorption of glucose, leading to an increase in urinary glucose excretion and hence lower blood glucose concentrations. Their action is independent of insulin, hence they reduce blood glucose concentrations without causing hypoglycaemia.^63^

They are recommended as co-first-line therapy in people with T2DM, or first-line therapy in patients unable to tolerate metformin, particularly those with atherosclerotic cardiovascular disease.^10^ Since 2013, there have been four SGLT-2i approved for use in the UK: dapagliflozin, canagliflozin, empagliflozin, and ertugliflozin.^42^

### Sodium glucose co-transporter-2 inhibitor use in heart failure and chronic kidney disease

In addition to their beneficial effects in T2DM, SGLT-2i have been shown to have cardioprotective and reno-protective effects, and have become treatment options in both heart failure and chronic kidney disease, irrespective of the presence of T2DM.^42^^,^^64^

### Sodium glucose co-transporter-2 inhibitor and diabetic ketoacidosis

One of the perioperative risks associated with SGLT-2i is the incidence of DKA. Owing to their mechanism of action, the DKA can be characterised by an increased ketone concentration, with normal, near normal, or increased blood glucose concentrations (usually <14 mmol L^−1^), termed euglycaemic DKA.^64^ With regards to people undergoing emergent surgery or those with critical illness on SGLT2i, those who have had a transition from insulin (or lowering of insulin doses and start or increase in SGLT2i), there is an increased risk for euglycaemic DKA. Restricted food intake or dehydration (i.e. perioperative fasting) and increased insulin requirements due to acute illness or surgery can also predispose people with diabetes to developing DKA.^65^^,^^66^ Emergency surgery is a particular risk factor, accounting for a quarter of case reports of DKA in a 2022 systematic review, that may be further complicated by decreased oral intake, dehydration, and infection in this group of patients.^67^

### Perioperative management of sodium glucose co-transporter-2 inhibitor inhibitors

The US Food and Drug Administration recommends SGLT-2i should be withheld 3 days before operation (canagliflozin, dapagliflozin, and empagliflozin) and 4 days before operation for ertugliflozin.^30^ This has been reflected in guidance from the American Diabetes Association and the Canadian Standards for Perioperative/Periprocedure Glycemic Management Expert Consensus Panel.^27^^,^^29^ Australian guidelines recommend withholding SGLT-2i 2 days before surgery (including procedures requiring bowel preparation), except for patients undergoing minor surgery, where the guidance is to omit on the morning of surgery only. In addition, the guidance states that consideration should be given to adjusting other glucose-lowering agents while SGLT-2i are withheld.^28^

In the UK, the CPOC recommends withholding SGLT-2i the day before and the day of surgery.^23^ On the basis that the majority of people are likely to take their SGLT-2i in the morning, this advice gives a cessation period of 48–54 h; this allows for an appropriate preoperative cessation period to reduce the risk of DKA whilst balancing the risk of worsening glycaemic control.^64^ The CPOC guidance advocates for appropriate perioperative monitoring for DKA (i.e. blood glucose and blood ketone concentrations), to mitigate the potential risks, which reflects the advice of the UK Medicines and Healthcare Products Regulatory Agency and Australian guidance.^23^^,^^28^^,^^65^

Seki and colleagues^67^ conducted a systematic review of case reports for SGLT-2i-associated perioperative DKA and found no cases of DKA with >2-day preoperative cessation period. Hence, there are currently no validated studies to support the longer cessation period advocated by some guidelines.^67^ In addition, the longer preoperative cessation times do not consider the potential risks of worsening glycaemic control, risks of cardiovascular and renal harm, or risks of perioperative insulin use.^64^ There has been one small RCT that has looked at continuation *vs* discontinuation of oral anti-diabetes agents perioperatively in the ambulatory setting. Although it found that continuation of oral anti-diabetes agents achieved better blood glucose concentrations, the study did not address the potential risks associated with euglycaemic DKA, hence cannot be used to inform practise.^18^

Special consideration should be given to those who require bowel preparation before operation or those required to follow a reduced calorie diet before their surgical procedure, such as in bariatric surgery. A longer period of drug cessation may be necessary and usually coincides with the reduced food intake.^23^^,^^32^ People undergoing bariatric surgery are often advised to reduce their calorie intake before their surgery (in some cases as low as 800 kcal per day), which is often a significant reduction in their usual daily calorie consumption, thus increasing the risk of DKA in these patients.^32^ There is currently no consensus guidance for the management of SGLT-2i in patients with a reduced calorie intake and it is recommended that individual centres ensure that they have their own agreed in-house guidelines.^23^

Currently, there are limited data to advise on the perioperative continuation/cessation of these medicines in patients who require emergency surgery. The UK CPOC advises that they should be held on admission, and CBG and capillary ketone concentrations should be monitored.^23^ However, a recently published study of 34 671 people with type 2 diabetes who underwent emergency surgery demonstrated that the preoperative use of SGLT2i was not associated with an increased risk for postoperative DKA compared with those not using SGLT2is.^68^ In addition to suggesting that there is a need to revisit the whole guidance on perioperative cessation of SGLT2i in individuals requiring elective surgery, it also suggests that in those undergoing elective surgery who have not stopped their SGLT2i medications, postponing the surgery and relisting them once they have stopped are probably unnecessary.^68^ A more appropriate response would be to undertake shared decision making about proceeding or postponing surgery and undertake appropriate glucose and ketone monitoring if the shared decision is to proceed with surgery.

In emergency surgery, where there may not be the time to appropriately hold SGLT-2i, they should be held on admission, and an appropriate CBG and capillary ketone concentration monitoring regimen be instituted.^23^

Combination products containing an SGLT-2i with either metformin or a DPP-4 inhibitor are available.^42^ Because of the potential prolonged cessation time perioperatively of SGLT-2i, consideration should be given to prescribing the components of these combination products as separate medicines perioperatively to reduce the potential risks associated with loss of glycaemia control.

### Postoperative management

The Australian guidelines recommend that SGLT-2i are not recommenced until a minimum of 2 days after major surgery and the day after minor surgery.^28^ Other guidance suggests that SGLT-2i can be restarted once normal oral intake has been established and ketone levels are normal, and the individual is due to leave hospital.^23^^,^^28^^,^^29^ However, it should be considered that the in-hospital use of SGLT2i has yet to be convincingly shown to be safe.^69^

After bariatric surgery, people with T2DM may have a rapid decrease in their blood glucose concentrations, meaning anti-diabetes agents may need to be reviewed or even stopped.^32^ This is of particular importance with patients on SGLT-2i as there have been cases reported of post-bariatric surgery DKA where SGLT-2i were implicated.^70^

## General considerations in the management of non-insulin diabetes medicines in critical care

Critical illness and resultant organ system dysfunction often result in significant alterations in the pharmacokinetics and pharmacodynamics of routinely administered medicines. Renal impairment, and the need for renal replacement therapy, can alter the handling of renally cleared agents, whereas gastrointestinal dysfunction and ileus can lead to unpredictable absorption of orally administered agents. Further potential concerns arise in the critically ill, where skin hypoperfusion in the presence of hypotension, vasoconstrictor use, or peripheral oedema may result in the unpredictable absorption of injectable medications such as the GLP-1RAs. As such, the decision to continue an individual’s routine medicines must always be individualised by weighing the risks of potential toxicity (due to accumulation or a failure of renal or hepatic metabolism) or lack of effect (due to disordered absorption). In general, specific recommendations for most non-insulin diabetes medicines are lacking, with international guidance on glycaemic control in the critically ill focusing on insulin therapy.^71^ In each of the following discussions, known pharmacokinetic, pharmacodynamic, and safety considerations for individual agents of relevance to critical care are highlighted.

### Dose modification of insulin secretagogues (sulfonylureas and meglitinides) in critical care

Because of the mode of action of these drugs, the risk of hypoglycaemia is increased in the presence of impaired enteral absorption, renal, and hepatic impairment.^42^ This means these agents should normally be stopped in critical illness. Additionally, the manufacturers of repaglinide advise that its hypoglycaemic action may be prolonged in individuals receiving clarithromycin, itraconazole, and clopidogrel that may be co-administered to critically ill patients.^42^

### Dose modification of metformin in critical care

Metformin has the least impact on basal lactate metabolism within the biguanide class; however, its effect may be significant in situations where other factors disturb the balance between lactate clearance and production.^72^ This may occur where tissue hypoperfusion is present. In addition, impaired renal function could lead to accumulation and increased plasma concentrations of metformin.^72^

Although many studies observe no increased risk of lactic acidosis with metformin, this may reflect study inclusion criteria.^72^ The clinical course of suspected cases of MALA relates to lactate concentrations with significant mortality being reported and many people requiring renal replacement therapy.^73^^,^^74^ In line with potential pathophysiology, the literature highlights that many cases are associated with acute intercurrent illness, deteriorating renal function, or metformin overdose. As such, it is recommended to discontinue metformin in the critically ill or those with risk factors for dehydration or deteriorating renal function.^72^^,^^73^^,^^75^

### Dose modification of incretin agonists in critical care

Currently, there is no specific advice on dose modification of incretin agonists in critical care. However, because of their known side-effects of reduced gastric emptying, and interference with the gastrointestinal tract, it would be prudent to withhold these medicines during critical illness. Importantly, the prolonged half-lives of these agents mean effects may persist for some time after their discontinuation. These agents are also associated with side-effects in multiple organ systems including acute pancreatitis and acute renal failure.^76^

### Dose modification of dipeptidyl peptidase-4 inhibitors in critical care

Like the incretin agonists, there is no specific advice on dose modification of DPP-4 inhibitors in critical care. However, apart from linagliptin, these agents are renally excreted and so dose modification is required with renal impairment. These medicines also carry a small risk of pancreatitis. There are additional concerns for an increased risk of heart failure with saxagliptin, although data suggest this is not a class effect.^61^^,^^77^ Subgroup analysis suggested this risk may be higher in those with impaired renal function.^77^ Thus, a prudent course of action is to withhold these medicines in critical care.

### Dose modification of sodium glucose co-transporter 2 inhibitors in critical care

The concerns around DKA is evident from randomised controlled trial data on the use of SGLT-2i and, alongside surgical patients, was more common in those with acute illness.^78^ As such, current international guidance is to discontinue these medicines during critical illness with frequent assessment of ketone levels.

### Dose modification of thiazolidinediones in critical care

These agents can predispose to fluid retention and may precipitate heart failure.^42^^,^^79^ Thus, their use in a critical care setting, where fluid overload is already common, is of concern.

### Dose modification of acarbose in critical care

Acarbose should normally be discontinued given its mode of action and contraindications in the setting of severe hepatic impairment and renal impairment (eGFR <25 ml/min/1.73 m^2^). Specific gastrointestinal contraindications also exist, including obstruction (or risk of), inflammatory bowel disease, or ulceration.^42^ Thus, specific care should be taken in the critically ill surgical patient.

## Ensuring safe use of non-insulin medicines for diabetes mellitus in the perioperative period

‘Sick day rules’ are intended to prevent complications, such as DKA, hyperosmolar hyperglycaemic state, and acute kidney injury. If a medicine is continued during concurrent illness whilst in the community, a decision should be made on whether it should be continued in the perioperative period.^80^ Dehydration increases the risk of lactic acidosis with metformin. Continuation of sulfonylureas whilst a person is unable to eat or drink is associated with an increased risk of hypoglycaemic episodes. SGLT-2 inhibitors are associated with ketoacidosis. Using the principles of ‘sick day rules’, non-insulin medicines for diabetes mellitus should be stopped and restarted when the person is well (normally after 24–48 h of eating and drinking normally). People with diabetes should be provided with written information about sick day rules in case they develop an acute illness on discharge.^23^

## Conclusions

T2DM affects approximately a quarter of the general surgical population, with the prevalence in some surgical populations (e.g. cardiac surgery) being >35%. Indiscriminate perioperative cessation of a person’s non-insulin medicines with resultant loss of glycaemic control cannot be advocated, because this may lead to harm from either hyperglycaemia or harm from the use of exogenous insulin.^7^ Thus, there is a need to modify the person’s diabetes medicines when it is safe and practical to do so. There is an absence of prospective randomised trials to advise on the most appropriate form of modification; thus, practitioners need to rely on pharmacological principles. This narrative review describes the mechanisms of action, and the perioperative risks associated with each class of non-insulin diabetes medicines, so that practitioners can apply first principles for a rationale for the perioperative management of the non-insulin diabetes medicines.

## Authors’ contributions

Initial idea for the manuscript: KD, NAL

Wrote early drafts and final versions of the manuscript: all authors

Read and approved the final version: all authors

## Declaration of interest

The authors declare that they have no conflicts of interest.
